# Neurodevelopmental Trajectories and Clinical Profiles in a Sample of Children and Adolescents With Early- and Very-Early-Onset Schizophrenia

**DOI:** 10.3389/fpsyt.2021.662093

**Published:** 2021-09-16

**Authors:** Maria Pontillo, Roberto Averna, Maria Cristina Tata, Fabrizia Chieppa, Maria Laura Pucciarini, Stefano Vicari

**Affiliations:** ^1^Child and Adolescent Neuropsychiatry Unit, Department of Neuroscience, Children Hospital Bambino Gesù, Istituto di Ricovero e Cura a Carattere Scientifico (IRCCS), Rome, Italy; ^2^Department of Life Sciences and Public Health, Catholic University of the Sacred Heart, Rome, Italy

**Keywords:** schizophrenia, neurodevelopmental disorders, early onset psychosis, children, adolescents

## Abstract

Schizophrenia before the age of 18 years is usually divided into two categories. Early-onset schizophrenia (EOS) presents between the ages of 13 and 17 years, whereas very-early-onset schizophrenia (VEOS) presents at or before the age of 12 years. Previous studies have found that neurodevelopmental difficulties in social, motor, and linguistic domains are commonly observed in VEOS/EOS patients. Recent research has also shown a high prevalence of neurodevelopmental disorders (e.g., intellectual disability, communication disorders, autism spectrum disorder, neurodevelopmental motor disorders) in VEOS/EOS patients, indicating genetic overlap between these conditions. These findings lend support to the neurodevelopmental continuum model, which holds that childhood neurodevelopmental disorders and difficulties and psychiatric disorders (e.g., schizophrenia) fall on an etiological and neurodevelopmental continuum, and should not be considered discrete entities. Based on this literature, in this study we focused on the overlap between neurodevelopmental disorders and schizophrenia investigating, in a large sample (*N* = 230) of VEOS/EOS children and adolescents, the clinical differences, at the onset of psychosis, between VEOS/EOS with neurodevelopmental disorder or neurodevelopmental difficulties and VEOS/EOS with no diagnosed neurodevelopmental disorder or neurodevelopmental difficulties. The findings showed that, in children and adolescents with a neurodevelopmental disorder or neurodevelopmental difficulties, psychosis onset occurred at an earlier age, was associated with more severe functional impairment (e.g., global, social, role), and was characterized by positive symptoms (e.g., grandiose ideas, perceptual abnormalities, disorganized communication) and disorganized symptoms (e.g., odd behavior or appearance, bizarre thinking). Instead, in children and adolescents without a neurodevelopmental disorder or neurodevelopmental difficulties, psychosis onset was mainly characterized by negative symptomatology (e.g., social anhedonia, avolition, expression of emotion, experience of emotions and self, ideational richness). Given these differences, the presence of a neurodevelopmental disorder or neurodevelopmental difficulties should be carefully investigated and integrated early into the assessment and treatment plan for VEOS/EOS patients.

## Introduction

Among children, schizophrenia is a rare neuropsychiatric disorder. The best prevalence estimates for schizophrenia in patients younger than 15 years is 0.05%, and only 2% of adult patients are estimated to have experienced the onset of their psychosis before the age of 13 years. According to the data and criteria provided by the National Institute of Mental Health (NIMH), in childhood and adolescence, two types of schizophrenia are commonly described, depending on the age of onset: early-onset schizophrenia (EOS), which presents between the ages of 13 and 17 years; and very-early-onset schizophrenia (VEOS), which presents at or before the age of 12 years. Although they are less common than adult-onset schizophrenia (AOS, presenting from the age of 17 years), VEOS and EOS tend to be more severe and disabling (1, 2). Concerning their clinical profile, both show higher rates of auditory hallucinations (3), negative symptoms, and bizarre behavior (4), as well as more severe neurocognitive difficulties (5), relative to AOS. Evidence of more severe neurocognitive difficulties among VEOS/EOS patients has been provided by anatomical brain MRI studies showing a greater progressive loss of cortical gray matter (6, 7) and progressive increases in ventricular volume (7) over time. In addition, compared with AOS patients, VEOS/EOS patients usually experience a longer duration of untreated psychosis (DUP) (8), worse outcomes (9), and more premorbid neurodevelopmental disorders (1).

Previous studies (10) have shown that premorbid neurodevelopmental difficulties are frequently present in children and adolescents who develop schizophrenia, especially those who experience an early onset of psychotic symptoms (e.g., VEOS patients). Within the NIMH cohort, Driver et al. (1, 11) observed that 67% of VEOS patients registered premorbid social, motor, language, and learning difficulties. This finding supports the results obtained by the same research group on a cohort of 118 participants with childhood-onset schizophrenia (11), of whom 55% (*n* = 65) showed premorbid academic difficulties, 72% (*n* = 85) had premorbid social difficulties, and 44% (*n* = 52) had premorbid motor difficulties; in addition, 20% (*n* = 24) screened positive for a pervasive developmental disorder (e.g., autism, Asperger syndrome) according to DSM-IV-TR criteria. Building on these findings, other longitudinal studies (12) have shown an overlap between early autistic symptoms and psychotic symptoms during childhood and adolescence. Specifically, in one study, 20–50% of VEOS participants were found to meet the criteria for premorbid autism spectrum disorder, according to DSM-5 criteria. Finally, some studies (11, 13, 14) have found the outcome and prognosis of VEOS/EOS patients to be positively correlated with the presence and severity of premorbid difficulties. Kincaid et al. (15) reported that VEOS/EOS patients with autistic features had higher symptom severity and poorer long-term outcomes compared with VEOS/EOS patients without autistic symptoms. Overall, these findings lend support to the neurodevelopmental continuum model, which holds that childhood neurodevelopmental difficulties and psychiatric disorders (e.g., schizophrenia) fall on an etiological and neurodevelopmental continuum, and should not be considered discrete entities (16). Recent studies (16, 17) have indicated a genetic overlap between schizophrenia and other syndromes that commonly arise during the developmental period. In the DSM-5, these syndromes are classified as “neurodevelopmental disorders,” and include intellectual disability (ID), communication disorders (CDs), autism spectrum disorder (ASD), neurodevelopmental motor disorders (including tic disorders), and specific learning disorders (SLDs).

Over the years, many empirical studies have shown that childhood neurodevelopmental disorders such as ID, ASD, and ADHD share specific genetic risk alleles, both with each other and with psychiatric disorders, particularly schizophrenia (16, 18). For example, Singh et al. (17) found that copy number variants associated with ID were significantly enriched in individuals with schizophrenia, concluding that many additional ID-related variants may be a risk factor for schizophrenia. Further support for this theory is provided by studies showing that individuals with ID have higher rates of schizophrenia than the general population (19, 20). More recently, Stanfield et al. (21) confirmed the relationship between ID and risk for psychotic positive symptoms in a large sample of adolescents (*N* = 168).

In addition, neuroimaging studies (22) on brain alteration in VEOS/EOS patients have found less intracranial, hippocampal, and amygdala volume and higher caudate, pallidum, and lateral ventricle volume compared with healthy controls. This result is relatively consistent with data reported for adult psychosis patients (23), with the exception of the lower intracranial volume (ICV). This lower ICV in VEOS/EOS patients, compared with adult psychosis patients, suggests more severe disruption of brain neurodevelopment and at an earlier age. Of note, previous studies have found lower ICV in children and adolescents with attention deficit hyperactivity disorder (ADHD) (24) and higher ICV in patients with ASD (22), highlighting the importance of considering ICV in neurodevelopmental disorder imaging studies. In addition, both children and adolescents with ADHD and adults suffering from schizophrenia and bipolar disorder have been found to have lower hippocampal volume (25, 26). Thus, lower hippocampal volume could be a feature of several neurodevelopment disorders, reflecting both shared and distinct illness mechanisms. Overall, these results provide further support for the neurodevelopmental continuum model, which considers neurodevelopmental disorders and psychiatric disorders (e.g., schizophrenia) diverse outcomes on the range of disrupted or deviant brain development (27).

However, most of the studies cited so far consider small samples of VEOS/EOS and do not deepen the differences in the clinical presentation of psychosis between VEOS/EOS with a neurodevelopmental disorder or neurodevelopmental difficulties and VEOS/EOS without a neurodevelopmental disorder or neurodevelopmental difficulties.

Studying clinical differences at the onset of psychosis in these clinical populations could allow us to better understand the overlap between neurodevelopmental disorders and schizophrenia, and thereby improve the diagnostic and treatment process. For example, previous studies (15) suggest that a longer duration of illness is associated with poorer long-term outcomes and higher symptom severity in VEOS/EOS with neurodevelopmental disorders or neurodevelopmental difficulties (i.e., autistic features). Therefore, an early screening of psychosis in children and adolescents with neurodevelopmental difficulties or diagnosed neurodevelopmental disorder could allow starting earlier both psychological and pharmacological treatments and possibly modify the clinical outcome. On this basis, the present study aimed to investigate, in a large sample (*N* = 230) of VEOS/EOS, the clinical differences, at the onset of psychosis, between VEOS/EOS with neurodevelopmental difficulties or diagnosed neurodevelopmental disorder as proposed in DSM-5 (ID, CDs, ASD, neurodevelopmental motor disorders, and SLDs) and VEOS/EOS with no diagnosed neurodevelopmental disorder or neurodevelopmental difficulties. In particular, we have focused on the age of psychosis onset, clinical profile at the onset of psychosis (e.g., positive, negative, disorganized, and general symptoms), and functional impairment at the onset of psychosis.

## Materials and Methods

### Participants and Procedure

The study participants were 230 children and adolescents who had been consecutively admitted to the Child and Adolescent Neuropsychiatry Unit of the Clinical and Research Hospital Bambino Gesù of Rome with a recent onset of psychosis between January 2018 and January 2020.

In particular, participants had been admitted to a specialized clinical service for clinical high risk and VEOS/EOS children and adolescents. We admitted help-seeking children and adolescents with a suspected prodromal condition or suspected presence of frank psychosis symptoms. At intake, a multi-specialized clinical team of child neuropsychiatrists and psychologists trained in developmental neuropsychology and psychopathology administered clinical evaluations based on present, past symptomatology, and neurodevelopmental history. This allows investigating the psychotic symptoms for which evaluation is required and the possible presence of a previous diagnosis of neurodevelopmental disorders or neurodevelopmental difficulties.

The single inclusion criterion was the presence of any schizophrenia spectrum disorder (according to the DSM-5) (28). The exclusion criteria were a past diagnosis of a psychotic disorder, the presence of a traumatic brain injury or neurological disorder, and current or previous drug or alcohol abuse. The participation rate was 77% of all consecutively admitted children and adolescents. Fifty-four patients (23%) were excluded on the basis of the exclusion criteria. None of the eligible patients refused to participate. The final sample was composed of 176 children and adolescents (67 female, 109 male). Patients experienced an onset of psychosis between the ages of 7 and 18 years (*M* = 14.69 years; *SD* = 2.19 years; median = 15 years).

All participants and their parents/legal guardians provided written informed assent and consent.

For all participants, the presence of a neurodevelopmental disorder or neurodevelopmental difficulties was ascertained from retrospective anamnestic data.

Table 1 presents the clinical criteria used to determine neurodevelopmental difficulties.

**Table 1:** Frequency and percentage of neurodevelopmental disorder (group 1) and neurodevelopmental difficulties (group 2).

**Variables**	**Group 1 *N* (%)**	**Group 2 *N* (%)**
**Neurodevelopmental disorder according to DSM-5**		
Intellectual disabilities	25 (38)	
Communication disorders	12 (18)	
Autism spectrum disorder	7 (11)	
Attention-deficit/hyperactivity disorder	21 (32)	
Specific learning disorder	18 (28)	
Motor disorders	6 (9)	
**Neurodevelopmental difficulties**		
Difficulties in psychomotor development: - *Unsupported sitting >8 months* - *Walking >18 months*		9 (24)
Language difficulties: - *First word >24 months* - *First 2/3 word phrases >36 months*		15 (39)
Reading/writing difficulties: - *School or parent report confirmed reading, writing, and calculation difficulties*		9 (24)
Social difficulties: - *Lack of reciprocal social communication* - *Failure to regulate gaze, facial expression, posture* - *Lack of imaginative or imitative play* - *Failure to make friends and share interests*		22 (58)

As result, participants were divided into the following groups:

- Group 1: VEOS/EOS patients with a diagnosed neurodevelopmental disorder [e.g., ID, CD, ASD, neurodevelopmental motor disorder (including tic disorder), SLD] before the onset of psychosis;
- Group 2: VEOS/EOS patients with neurodevelopmental difficulties (i.e., motor, linguistic, social difficulties) but no diagnosed neurodevelopmental disorder (28) before the onset of psychosis; and
- Group 3: VEOS/EOS patients with no diagnosed neurodevelopmental disorder or neurodevelopmental difficulties before the onset of psychosis.

### Clinical Assessment

Clinical assessment was conducted on the entire sample (*N* = 230) by a neuropsychiatrist and two psychologists. All the clinicians had been trained in the application of structured and semi-structured neuropsychiatric and psychopathological diagnostic tools, and were specialized in the evaluation of psychotic symptoms in children and adolescents, with and without a comorbid neurodevelopmental disorder.

Mental disorders were assessed using the Schedule for Affective Disorders and Schizophrenia for School Aged Children Present and Lifetime Version DSM-5 (K-SADS-PL DSM-5). K-SADS-PL DSM-5 investigates the possible presence of psychopathological disorders according to DSM-5 (including schizophrenia spectrum disorders) (29). The K-SADS-PL DSM-5, as proposed in the instrument manual by Kaufman et al. (29), provides as a source of information not only the child/adolescent but also the parent. In addition, for some particular cases (i.e., ID), the parent is considered the main source of information with respect to the child.

Psychotic symptoms were indexed using the Structured Interview for Psychosis-Risk Syndrome (SIPS/SOPS) (30). The SIPS/SOPS measures four symptom dimensions: *positive symptoms* (i.e., unusual thought content/delusional ideas, suspiciousness/persecutory ideas, grandiose ideas, perceptual abnormalities/hallucinations, disorganized communication), *negative symptoms* (i.e., social anhedonia, avolition, expression of emotion, experience of emotions and self, ideational richness, occupational functioning), *disorganized symptoms* (i.e., odd behavior or appearance, bizarre thinking, trouble with focus and attention, impairment in personal hygiene), and *general symptoms* (i.e., sleep disturbance, dysphoric mood, motor disturbances, impaired tolerance to normal stress). These dimensions are assessed according to the presence, duration, and severity of specific experiences and behaviors, with each item rated on a scale of 0 (*symptom absent*) to 6 (*extreme* symptom intensity, or *psychotic* for the Positive Symptom items).

SIPS/SOPS interviews were conducted by clinical psychologists and neuropsychiatrists trained by the main author of the factor-structure analysis study on the Italian version of the Scale of Prodromal Symptoms (SOPS) in comparison with the English version (31).

For children under the age of 13 years and for children and adolescents with ID, the K-SADS PL DSM-5 and the SIPS/SOPS interviews consult both the child and the parents. According to procedure used in previous studies (19, 21, 32, 33), K-SADS-PL DSM-5 and SIPS/SOPS interviews were completed face to face with children and adolescents with IDs supported by their caregiver or another appropriate person. Information was also collected from relatives separately.

Level of global functioning (referring to family, school, and social domains) was measured with the Childhood Global Assessment Scale (34). This scale assesses functional impairment due to neuropsychiatric disorders and produces a score ranging from 1 (*constant supervision*) to 100 (*functioning above the norm in all areas*). Social and role functioning were assessed using the Global Functioning: Social Scale (GF: Social) (35) and the Global Functioning: Role Scale (GF: Role) (36) to obtain differential measures of functioning. GF: Social investigated the quantity and the quality of peer relationship, involvement with family members, and level of peer conflict. GF: Role investigated the level of performance in school, work, or at home. These scales are based on 10 criteria of functioning, assessed from 1 (*extreme social isolation* or *role impairment*, respectively) to 10 (*higher interpersonal* and *role functioning*, reflectively). Both scales provide indications of current functioning, lowest functioning in the past year, and highest functioning in the past year.

Neurocognitive functioning (IQ) was measured with the Wechsler Intelligence Scales (WISC-IV: Wechsler Intelligence Scale for Children; WAIS-IV: Wechsler Adult Intelligence Scale) (37, 38).

Finally, we documented the presence of substance use and diagnosed psychosis in patients' first- and second-degree relatives.

### Statistical Analyses

Statistical analyses were performed with Stata for Windows (StataCorp LLC, College Station, Texas; version 13.0 released in 2013). One-way analyses of variance (*F*-tests or ANOVAs) were used to test the independence between continuous response variables and the categorical explanatory variable (group variable). Row scores for each measure were considered.

*Post-hoc* analyses were performed to determine Bonferroni CIs (95%) and establish differences between means. Associations between group, sex, and type of substance use (i.e., nicotine, alcohol, cannabis) were examined using χ^2^-tests.

## Results

### Sample Characteristics

The final sample (*N* = 176; mean age of psychosis onset = 14.69 years, *SD* = 2.19; male: *n* = 109, 62%) was divided into three groups. Group 1 was composed of 65 (37%) VEOS/EOS subjects (male: *n* = 43; 66%) with a diagnosed neurodevelopmental disorder. Group 2 consisted of 38 (22%) VEOS/EOS subjects (male: *n* = 25; 66%) with neurodevelopment difficulties.

Finally, group 3 included 73 (41%) VEOS/EOS subjects (male: *n* = 41; 56%) with neither neurodevelopment difficulties nor a diagnosed neurodevelopmental disorder.

There were no significant differences in sex (Pearson χ^2^ = 1.7612, *p* = 0.415) between groups (total sample: 62% male).

Table 1 reports the percentage frequency of neurodevelopmental disorders in group 1 and neurodevelopmental difficulties in group 2.

Table 2 reports the frequency and percentage frequency of diagnosed schizophrenia spectrum disorders and a family history of psychiatric illness and/or substance use, for each group.

**Table 2:** Socio-demographic and clinical data scores separately for three groups.

**Variables**	**Group 1 *N* (%)**	**Group 2 *N* (%)**	**Group 3 *N* (%)**	**Statistics**
Male gender	43 (66)	25 (66)	41 (56)	χ^2^ = 1.7612 *p* = 0.415
Nicotine use	1 (2)	1 (3)	5 (7)	χ^2^ = 2.6666 *p* = 0.615
Alcohol use	0 (0)	0 (0)	3 (4)	
Cannabis use	9 (14)	3 (8)	17 (23)	
First-degree relative with psychotic disorder	1 (2)	4 (11)	1 (2)	
Second-degree relative with psychotic disorder	5 (8)	5 (13)	5 (7)	
**Schizophrenia spectrum disorders according to DSM-5**				
Delusional disorder	0 (0)	0 (0)	3 (4)	
Brief psychotic disorder	20 (31)	8 (21)	21 (29)	
Schizophreniform disorder	10 (15)	5 (13)	22 (30)	
Schizophrenia	32 (49)	24 (63)	21 (29)	
Schizoaffective disorder	3 (5)	1 (3)	6 (8)	

No significant differences were found in substance use (Pearson χ^2^ = 2.6666, *p* = 0.615) between groups.

### Comparison Between Groups

#### Age of Psychosis Onset, Number of Hospitalizations, IQ, and Functioning

As shown in Table 3, significant differences between three groups were found in age of psychosis onset [*F*_(2, 173)_ = 6.70, *p* = 0.0016]. Bonferroni *post-hoc* analyses showed that groups 1 and 2 presented a younger age of psychosis onset than group 3 (Gr1 vs. Gr2: *p* = 1.000; Gr1 vs. Gr3: *p* = 0.002; Gr2 vs. Gr3: *p* = 0.046).

**Table 3:** Group differences on age of psychotic onset, number of hospitalizations, IQ, and functioning.

**Variables**	**Group 1 Mean (*SD*)**	**Group 2 Mean (*SD*)**	**Group 3 Mean (*SD*)**	***F***	***p*-value**
Age of psychosis onset (years)	14.12 (2.54)	14.34 (2.25)	15.38 (1.58)	6.70	0.0016^*^
Number of hospitalizations	1.31 (1.58)	1.71 (3.10)	1.37 (0.99)	0.62	0.5387
IQ	75.81 (18.90)	88.18 (11.09)	90.21 (12.83)	17.19	0.0000^**^
C-GAS	40.83 (7.67)	40.34 (6.35)	45.72 (6.37)	11.69	0.0000^**^
GF: Role	3.16 (0.83)	3.18 (0.69)	3.67 (0.68)	9.40	0.0001^**^
GF: Social	3.16 (0.83)	3.18 (0.69)	3.67 (0.68)	9.40	0.0001^**^

* *p ≤ 0.005 and*

** *p ≤ 0.0001*.

*IQ, intelligence quotient; C-GAS, Children's Global Assessment Scale; GF: Social, Global Functioning: Social Scale; GF: Role, Global Functioning: Role Scale*.

Regarding number of hospitalizations, no significant differences were found between groups [*F*_(2, 173)_ = 0.62, *p* = 0.5387].

IQ significantly differed between groups [*F*_(2, 173)_ = 17.19, *p* = 0.000]. Bonferroni *post-hoc* analyses showed that group 1 had lower scores than groups 2 and 3 (Gr1 vs. Gr2: *p* = 0.000; Gr1 vs. Gr3: *p* = 0.000; Gr2 vs. Gr3: *p* = 1.000).

There were also significant differences between groups with respect to global, social, and role functioning.

Bonferroni *post-hoc* analyses showed that groups 1 and 2 presented with significantly worse global functioning measured by C-GAS [*F*_(2, 173)_ = 11.69, *p* = 0.000] compared with group 3 (Gr1 vs. Gr2: *p* = 1.000; Gr1 vs. Gr3: *p* = 0.000; Gr2 vs. Gr3: *p* = 0.000). With respect to GF-Role and GF-Social, similar results were found, with groups 1 and 2 presenting significant social and role impairment [*F*_(2, 173)_ = 9.40, *p* = 0.0001] relative to group 3 (Gr1 vs. Gr2: *p* = 1.000; Gr1 vs. Gr3: *p* = 0.000; Gr2 vs. Gr3: *p* = 0.004).

These significant group differences in global [*F*_(2, 173)_ = 13.71, *p* = 0.000], role [*F*_(2, 173)_ = 10.30, *p* = 0.0001], and social [*F*_(2, 173)_ = 10.30, *p* = 0.0001] functioning were maintained when covariate IQ was added. Significant group differences in global [*F*_(2, 173)_ = 8.12, *p* = 0.0004], role [*F*_(2, 173)_ = 5.35, *p* = 0.0055], and social [*F*_(2, 173)_ = 5.35, *p* = 0.0055] functioning were also maintained when the covariate number of diagnoses was added.

#### SIPS/SOPS Psychotic Symptoms Dimensions

Table 4 reports significant group differences for each SIPS/SOPS symptom dimension.

**Table 4:** Group differences on SIPS/SOPS.

**Variables**	**Group 1 Mean (*SD*)**	**Group 2 Mean (*SD*)**	**Group 3 Mean (*SD*)**	***F***	***p*-value**
**SIPS positive items**					
P1: Unusual thought content/delusional ideas	5.18 (1.43)	5.23 (1.34)	5.45 (1.16)	0.79	0.4573
P2: Suspiciousness/persecutory ideas	4.35 (1.97)	4.39 (1.79)	4.69 (1.91)	0.64	0.5279
P3: Grandiose ideas	2.03 (1.80)	2.13 (1.72)	1.34 (1.60)	3.91	0.0218^*^
P4: Perceptual abnormalities/hallucinations	4.81 (1.75)	4.57 (1.60)	3.91 (2.28)	3.79	0.0245^*^
P5: Disorganized communication	4.55 (1.40)	3.94 (1.94)	3.83 (2.02)	2.98	0.0496^*^
SIPS positive total score	20.93 (3.62)	20.28 (4.59)	19.24 (4.72)	2.68	0.0711
**SIPS negative items**					
N1: Social anhedonia	4.29 (1.68)	4.89 (1.68)	5.31 (1.43)	7.18	0.0010^*^
N2: Avolition	3.92 (1.73)	3.52 (2.12)	4.80 (1.34)	8.70	0.0003^*^
N3: Expression of emotion	3.46 (1.59)	4.26 (1.40)	5.00 (1.36)	19.05	0.0000^**^
N4: Experience of emotions and self	3.12 (1.70)	4.00 (1.33)	4.90 (1.39)	24.14	0.0000^**^
N5: Ideational richness	3.60 (1.59)	4.84 (1.28)	4.49 (1.39)	10.69	0.0000^**^
N6: Occupational functioning	5.41 (1.05)	5.78 (0.47)	5.52 (1.24)	1.53	0.2194
SIPS Negative total score	23.81 (6.99)	27.31 (5.46)	30.04 (7.02)	14.82	0.0000^**^
**SIPS disorganized items**					
D1: Odd behavior or appearance	4.70 (1.53)	4.39 (1.51)	3.87 (1.97)	4.04	0.0193^*^
D2: Bizarre thinking	4.92 (1.58)	4.73 (1.68)	4.02 (2.16)	4.30	0.0150^*^
D3: Trouble with focus and attention	3.81 (1.50)	4.10 (1.20)	3.60 (1.70)	1.35	0.2621
D4: Impairment in personal hygiene	3.43 (1.66)	3.50 (1.79)	2.93 (1.58)	2.15	0.1196
SIPS Disorganized total score	18.87 (3.74)	16.73 (4.58)	14.43 (5.20)	5.81	0.0036^*^
**SIPS general items**					
G1: Sleep disturbance	3.93 (1.88)	4.36 (1.73)	4.56 (1.78)	2.08	0.1284
G2: Dysphoric mood	4.52 (1.64)	4.78 (1.59)	4.76 (1.64)	0.49	0.6154
G3: Motor disturbances	2.55 (1.87)	2.78 (1.86)	2.83 (1.91)	0.41	0.6614
G4: Impaired tolerance to normal stress	4.29 (1.89)	4.84 (1.76)	4.31 (1.77)	1.31	0.2726
SIPS general total score	15.30 (4.78)	16.78 (4.21)	16.47 (5.00)	1.53	0.2199

* *p ≤ 0.05 and*

** *p ≤ 0.0001*.

*SIPS, Structured Interview for Psychosis-Risk Syndrome*.

##### Positive Symptoms

Significant group differences were found in grandiose ideas [*F*_(2, 173)_ = 3.91, *p* = 0.0218], perceptual abnormalities/hallucinations [*F*_(2, 173)_ = 3.79, *p* = 0.0245], and disorganized communication [*F*_(2, 173)_ = 2.98, *p* = 0.0496]. Bonferroni *post-hoc* analyses were conducted; comparisons of all groups were made. Regarding grandiose ideas, group 1 reported major mean score compared with group 3 (Gr1 vs. Gr3: *p* = 0.050). All other comparisons were not significant (Gr1 vs. Gr2: *p* = 1.000; Gr2 vs. Gr3: *p* = 0.066). Concerning perceptual abnormalities/hallucinations, group 1 reported major score compared with group 3 (Gr1 vs. Gr3: *p* = 0.025). All other comparisons were not significant (Gr1 vs. Gr2: *p* = 1.000; Gr2 vs. Gr3: *p* = 0.285). Finally, with respect to disorganized communication, group 1 reported major score compared with group 3 (Gr1 vs. Gr3: *p* = 0.049). All other comparisons were not significant (Gr1 vs. Gr2: *p* = 0.302; Gr2 vs. Gr3: *p* = 1.000).

No significant group differences were found in unusual thought content/delusional ideas [*F*_(2, 173)_ = 0.79, *p* = 0.4573] or suspiciousness/persecutory ideas [*F*_(2, 173)_ = 0.64, *p* = 0.5279]. Globally, no significant differences were found in regards to the SIPS total score for positive symptoms [*F*_(2, 173)_ = 2.68, *p* = 0.0711].

##### Negative Symptoms

With respect to negative symptoms, significant group differences were found in social anhedonia [*F*_(2, 173)_ = 7.18, *p* = 0.0010], avolition [*F*_(2, 173)_ = 8.70, *p* = 0.0003], expression of emotion [*F*_(2, 173)_ = 19.05, *p* = 0.0000], experience of emotions and self [*F*_(2, 173)_ = 24.14, *p* = 0.0000], and ideational richness [*F*_(2, 173)_ = 10.69, *p* = 0.0000]. Bonferroni *post-hoc* analyses were conducted; comparisons of all groups were made. Concerning social anhedonia, group 3 reported major score compared with group 1 (Gr1 vs. Gr3: *p* = 0.001). All other comparisons were not significant (Gr1 vs. Gr2: *p* = 0.193; Gr2 vs. Gr3: *p* = 0.560). Regarding avolition, group 3 reported major score compared with groups 1 and 2 (Gr1 vs. Gr3: *p* = 0.007; Gr2 vs. Gr3: *p* = 0.001). All other comparisons were not significant (Gr1 vs. Gr2: *p* = 0.746). With respect to expression of emotion, group 3 reported major score compared with groups 1 and 2 (Gr1 vs. Gr2: *p* = 0.024; Gr1 vs. Gr3: *p* = 0.000; Gr2 vs. Gr3: *p* = 0.038). Also, in experience of emotions and self, group 3 reported major score compared with groups 1 and 2 (Gr1 vs. Gr2: *p* = 0.014; Gr1 vs. Gr3: *p* = 0.000; Gr2 vs. Gr3: *p* = 0.009).

Finally, regarding ideational richness, group 3 reported major score compared with group 1 (Gr1 vs. Gr3: *p* = 0.001). Also, group 2 reported major score compared with group 1 (Gr1 vs. Gr2: *p* = 0.000). Other comparison was not significant (Gr2 vs. Gr3: *p* = 0.694). No significant group differences were found with respect to occupational functioning [*F*_(2, 173)_ = 1.53, *p* = 0.2194].

Globally, significant differences were found for the SIPS total score for negative symptoms [*F*_(2, 173)_ = 14.82, *p* = 0.0000] with group 3 reporting major score compared with group 1 (Gr1 vs. Gr3: *p* = 0.000). Also, groups 1 and 2 differed significantly (Gr1 vs. Gr2: *p* = 0.034). All other comparisons were not significant (Gr2 vs. Gr3: *p* = 0.131).

##### Disorganized Symptoms

Significant group differences were found with respect to odd behavior or appearance [*F*_(2, 173)_ = 4.04, *p* = 0.0193], and bizarre thinking [*F*_(2, 173)_ = 4.30, *p* = 0.0150].

Bonferroni *post-hoc* analyses were conducted; comparisons of all groups were made.

Specifically, group 1 reported major score in odd behavior or appearance compared with group 3 (Gr1 vs. Gr3: *p* = 0.016). All other comparisons were not significant (Gr1 vs. Gr2: *p* = 1.000; Gr2 vs. Gr3: *p* = 0.409). Also, group 1 reported major score in bizarre thinking compared with group 3 (Gr1 vs. Gr3: *p* = 0.017). All other comparisons were not significant (Gr1 vs. Gr2: *p* = 1.000; Gr2 vs. Gr3: *p* = 0.178).

No significant group differences were found with respect to trouble with focus and attention [*F*_(2, 173)_ = 1.35, *p* = 0.2621], or impairment in personal hygiene [*F*_(2, 173)_ = 2.15, *p* = 0.1196]. Overall, significant group differences were found in the SIPS total score for disorganized symptoms [*F*_(2, 173)_ = 5.81, *p* = 0.0036]. In particular, group 1 reported major total score compared with groups 2 and 3 (Gr1 vs. Gr3: *p* = 0.006; Gr2 vs. Gr3: *p* = 0.039). All other comparisons were not significant (Gr1 vs. Gr2: *p* = 1.000).

##### General Symptoms

No significant group differences were found with respect to sleep disturbance [*F*_(2, 173)_ = 2.08, *p* = 0.1284], dysphoric mood [*F*_(2, 173)_ = 0.49, *p* = 0.6154], motor disturbances [*F*_(2, 173)_ = 0.41, *p* = 0.6614], or impaired tolerance to normal stress [*F*_(2, 173)_ = 1.31, *p* = 0.2726]. Overall, there were no significant group differences in the SIPS total score for general symptoms [*F*_(2, 173)_ = 1.53, *p* = 0.2199].

## Discussion

The main aim of the present study was to explore, in a large sample of VEOS/EOS, the clinical differences, at the onset of psychosis, between VEOS/EOS with neurodevelopmental difficulties or diagnosed neurodevelopmental disorder as proposed in DSM-5 (ID, CDs, ASD, neurodevelopmental motor disorders, and SLDs) and VEOS/EOS with no diagnosed neurodevelopmental disorder or neurodevelopmental difficulties.

We have focused on the age of psychosis onset, clinical profile at the onset of psychosis (e.g., positive, negative, disorganized, and general symptoms), and functional impairment at the onset of psychosis.

The first finding suggested that neurodevelopmental disorders may be common in our sample VEOS/EOS children and adolescents. Indeed, in our large sample (*N* = 176), 65 (37%) participants had a diagnosed neurodevelopmental disorder. Most frequently, the diagnoses were for ID (*n* = 25; 38%) and ADHD (*n* = 21; 32%). This finding is consistent with the results of previous studies showing that ID is common in individuals who develop schizophrenia (19, 32). Also, a history of ADHD symptoms is common in children and adolescents who develop schizophrenia (39), and ADHD is diagnosed in a high proportion of children at genetic risk for schizophrenia (40).

In addition, 22% of our participants had neurodevelopmental difficulties (e.g., motor, social, linguistic difficulties) that did not meet the criteria for a frank neurodevelopmental disorder. Specifically, 58% showed social difficulties, 39% showed language difficulties, 24% showed psychomotor difficulties, and 24% showed learning difficulties. These results are consistent with previous research finding that motor, linguistic, and social difficulties are present in children and adolescents who develop schizophrenia (1, 11).

Concerning the age of psychosis onset, in our sample, children and adolescents with a diagnosed neurodevelopmental disorder or neurodevelopmental difficulties showed an earlier onset of psychosis compared with participants with no neurodevelopmental diagnosis or difficulties. This result also aligns with the previous studies (10, 14, 41–43), which reported an association between earlier psychosis onset and the presence of language, motor, and social difficulties. For example, Petruzzelli et al. (44) found early onset of schizophrenia in 36 patients (age range: 7–17 years), of whom 70.6% presented neurodevelopmental difficulties, as well as difficulties in school learning or difficulties in sphincter control (enuresis).

Regarding clinical profile at the onset of psychosis, based on the SIPS/SOPS domains, we distinguished between positive symptoms, negative symptoms, disorganized symptoms, and general symptoms. Regarding positive symptoms, VEOS/EOS patients with a diagnosed neurodevelopmental disorder scored higher on grandiose ideas, perceptual abnormalities/hallucinations, and disorganized communication than VEOS/EOS patients with neurodevelopmental difficulties and VEOS/EOS patients with neither a neurodevelopmental disorder nor neurodevelopmental difficulties. The same pattern was found for disorganized symptoms, with VEOS/EOS patients with a diagnosed neurodevelopmental disorder scoring higher in odd behavior or appearance and bizarre thinking.

Regarding negative symptoms, VEOS/EOS patients with neither a neurodevelopmental disorder nor neurodevelopmental difficulties scored higher on social anhedonia, avolition, expression of emotion, experience of emotions and self, and ideational richness, relative to VEOS/EOS patients with a neurodevelopmental disorder or neurodevelopmental difficulties. Regarding general symptoms, we found no significant differences between groups.

Finally, regarding functional impairment at the onset of psychosis, in our sample, VEOS/EOS patients with a neurodevelopmental disorder or neurodevelopmental difficulties reported major global functional impairment, whereas VEOS/EOS patients without these two conditions did not. To understand the effect of neurodevelopmental disorders and difficulties on functioning in our VEOS/EOS sample, we also investigated two specific functioning domains: role and social. Both of these domains were found to be more compromised in VEOS/EOS patients with a neurodevelopmental disorder or neurodevelopmental difficulties than in VEOS/EOS patients with neither of these two conditions. We examined the relationship between neurodevelopmental disorders and difficulties and global, social, and role functioning, controlling for IQ (in the subgroup with neurodevelopmental disorders we included patients with ID) and number of neuropsychiatric disorders (neurodevelopmental disorders plus VEOS/EOS). The results showed that the poorer global, social, and role functioning of VEOS/EOS patients with a neurodevelopmental disorder or neurodevelopmental difficulties was not affected by any of these variables. In other words, neurodevelopmental disorders and difficulties tended to determine poorer global, social, and role functioning, regardless of cognitive functioning and the number of neuropsychiatric disorders.

Overall, based on our findings, we propose that the clinical picture of VEOS/EOS patients may differ at the onset of psychosis according to the presence (or lack) of a neurodevelopmental disorder or neurodevelopmental difficulties. In more detail, in children and adolescents with a neurodevelopmental disorder or neurodevelopmental difficulties, psychosis onset tends to occur at an earlier age and is associated with more severe (global, social, and role) functional impairment. In addition, in these patients, the onset of psychosis is likely to be characterized by positive symptoms (e.g., grandiose ideas, perceptual abnormalities, disorganized communication) and disorganized symptoms (e.g., odd behavior or appearance, bizarre thinking). Instead, in children and adolescents without a neurodevelopmental disorder or neurodevelopmental difficulties, the onset of psychosis is likely to be characterized by negative symptomatology (e.g., social anhedonia, avolition, expression of emotion, experience of emotions and self, ideational richness). These results should be replicated in future studies, with caution, to gain further information on the clinical and neurodevelopmental profiles of children and adolescents with suspected VEOS/EOS. Such research is essential for the early recognition of symptoms at the onset of psychosis and the preparation of a therapeutic program tailored to each patient.

The present research was the first study to explore the differences on clinical presentation of psychosis between VEOS/EOS with a neurodevelopmental disorder or neurodevelopmental difficulties and VEOS/EOS without a neurodevelopmental disorder or neurodevelopmental difficulties. Among the strengths that can be considered are the size of the sample and the age range (7–18 years) adequately representative of the developmental age. The research also considered a rich neurodevelopmental profile—considering both neurodevelopmental disorders (according to the DSM-5) and neurodevelopmental difficulties—and assessed psychotic symptoms (e.g., SIPS/SOPS) and level of functioning (e.g., GF: Role, GF: Social) at the onset of psychosis using semi-structured interviews. The study has several limitations. The first relates to the high prevalence of ID in the group with a neurodevelopmental disorder. This may have overestimated the presence of psychotic positive symptoms in this group. The second relates to the lack of data analysis about patients' pharmacological or psychosocial treatment before the onset of psychosis. The third relates to the lack, in the literature, of standardized structured or semi-structured interviews that can be used for the assessment of psychotic symptoms in children and adolescents with IDs. Future research should examine this aspect through longitudinal studies focused on the clinical overlap between neurodevelopmental disorders, neurodevelopmental difficulties, and VEOS/EOS.

## Data Availability Statement

The study data are not available due to ethical concerns. Patient privacy and security are protected, according to the ethical rules of our institutions and their restriction on data sharing.

## Ethics Statement

The studies involving human participants were reviewed and approved by Children Hospital Bambino Gesù. Written informed consent to participate in this study was provided by the participants' legal guardian/next of kin.

## Author Contributions

MP and RA conceived the research study. MCT and MLP collected data. MP and MCT contributed to the data analysis/interpretation. MP, MCT, FC, and MLP contributed to the writing of the article. MP, RA, and SV critically reviewed the final draft of the article. All authors contributed to the article and approved the submitted version.

## Conflict of Interest

The authors declare that the research was conducted in the absence of any commercial or financial relationships that could be construed as a potential conflict of interest.

## Publisher's Note

All claims expressed in this article are solely those of the authors and do not necessarily represent those of their affiliated organizations, or those of the publisher, the editors and the reviewers. Any product that may be evaluated in this article, or claim that may be made by its manufacturer, is not guaranteed or endorsed by the publisher.
